# Public perceptions of digital mental health awareness campaign in the Arab Gulf states: a qualitative thematic analysis

**DOI:** 10.3389/fpubh.2024.1477315

**Published:** 2024-12-13

**Authors:** Noura Alomair, Ghadah Alkhaldi, Norah M. Alsadhan, Rawan Alkasabi, Samah Alageel

**Affiliations:** ^1^Department of Community Health Sciences, College of Applied Medical Sciences, King Saud University, Riyadh, Saudi Arabia; ^2^Insurance Operations Policies Department, Insurance Authority, Riyadh, Saudi Arabia

**Keywords:** mental health, digital health, mental illness, culture, religion, stigma

## Abstract

Mental illness is a significant public health concern and a leading cause of disability worldwide. Research shows a lack of mental health knowledge and inappropriate practices in the Gulf Cooperation Council (GCC) states. Our study aimed to evaluate individuals’ perspectives on mental health by analyzing their responses to a digital campaign directed at GCC adolescents. We conducted a qualitative thematic analysis of comments in response to the Gulf Health Council’s mental health campaign. The campaign content was shared on four social media platforms: TikTok, YouTube, Instagram, and X. A total of 2,146 comments were included in the analysis. There was a widespread denial of the existence of mental illness. The comments revealed a lack of understanding and insufficient support for individuals dealing with mental health issues. Stigma and discrimination against people with mental illness were evident in the comments. The general perception was that individuals have control over their mental health, often associating mental illness with weakness and lack of willpower. Mental illness was believed to be caused by religious and moral shortcomings, and religion was viewed as the solution. Some comments highlighted the need to acknowledge mental illness and urged the development of strategies to promote mental health. Our research shows a lack of awareness, stigma, and inadequate resources for individuals dealing with mental health issues. It highlights the importance of addressing barriers to mental healthcare and increasing access to support. Interventions focusing on stigma reduction and promoting acceptance of mental health disorders are crucial and require collaborative efforts from various stakeholders.

## Introduction

In 2019, one in eight people was living with a mental health disorder globally (1). The rates of anxiety and depression increased dramatically in 2020 due to the global COVID-19 pandemic (1). The Middle East and North Africa (MENA) region is facing a significant burden of mental disorders (2, 3). It is estimated that around 15% of the population is affected by depression, while 10% are affected by general anxiety disorder. However, socio-cultural beliefs surrounding mental health in the MENA region may have led to under-reporting and under-diagnosis of mental health disorders. People with mental health issues often have limited access to adequate care, which can be attributed partly to the stigma and discrimination that they face, negatively impacting their experience (1).

The way mental health issues are perceived, diagnosed, and treated can differ significantly based on cultural beliefs, values, and practices (4). For example, some cultures consider mental illness as a spiritual issue or a punishment from God (5). In the context of the Gulf Cooperation Council (GCC) countries, spirituality and religiosity play a profound role in shaping perceptions of mental health (6). The strong influence of Islamic teachings in the region means that religious beliefs often intersect with health practices (7). This can lead to a dual effect: on the one hand, religion can provide a source of comfort, support, and community cohesion, promoting positive mental health outcomes through shared beliefs and practices (5). On the other hand, it can perpetuate misconceptions about the nature of mental illness, such as associating it with supernatural causes or viewing it as a result of insufficient faith or spiritual failings (5). These beliefs may lead to stigmatization, reluctance to seek professional help, and a preference for religious or traditional healing methods over medical treatment (6).

Studies conducted in the Arab states of the Gulf Cooperation Council (GCC) have revealed a limited level of mental health literacy and inappropriate mental health practices among both the general public and healthcare professionals (8). For instance, some people believe that a lack of religiosity causes mental illness (9). This belief, combined with the lack of knowledge, leads to stigmatization and negative attitudes toward people with mental health disorders (8, 9). Cultural beliefs toward mental health influenced healthcare professionals’ views about mental health disorders and contributed to the inadequate availability of mental healthcare services (8, 10). Some healthcare professionals in the GCC and MENA regions link mental illness with weakness and believe that mental health should be treated by seeking social and religious support (10, 11).

Several campaigns and interventions are in place worldwide to reduce stigma toward mental health disorders and promote mental health. Anti-stigma interventions using different media platforms (mass media and social media) can potentially improve people’s knowledge and attitudes toward mental health and encourage positive mental health practices (12). Although the effectiveness of digital interventions in supporting mental health has been established, these interventions must be personalized to the population’s needs, and access to them must be facilitated (13).

The Gulf Health Council (GHC) is active in the GCC region’s preventive, curative and rehabilitative health (14). One of the main objectives of the GHC is to spread culturally and religiously appropriate health awareness programs among GCC countries, citizens, and residents. The GCC region is part of the MENA region and includes Bahrain, Kuwait, Oman, Qatar, Saudi Arabia, and the United Arab Emirates. Islam is the predominant religion in the GCC region, with countries in the GCC region sharing similar languages (Arabic) and cultural practices.

In 2021, the Gulf Health Council (GHC) launched a comprehensive campaign aimed at improving mental health awareness among young individuals across the GCC region (15). The initiative sought to address the urgent mental health issues youth face by utilizing various resources. This included interactive online assessment tools and thorough educational content covering various mental health aspects. To make the message more relatable and impactful, the campaign featured compelling narratives from young people who shared their personal experiences with mental illness (16). These stories sought to foster a deeper understanding of mental health challenges and reduce stigma within the community. The campaign highlighted the importance of support systems, encouraged open conversations about mental well-being, and emphasized the necessity of seeking treatment.

It is essential to examine the unique responses of the Muslim and Arab communities concerning mental health discussions. By understanding their distinct perspectives and lived experiences, we can more effectively address mental health challenges within the GCC region. This understanding will enhance our approach and inform impactful public health interventions for a healthier future, combating stigma and fostering a supportive environment for mental well-being.

## Methods

This study aimed to investigate people’s opinions and views toward a mental health digital campaign targeted at adolescents and young adults in the GCC. The campaign was developed by a team of public health experts and content creators, and the content was produced in Arabic. In September 2021, the GHC launched a three-month mental health campaign to promote mental health awareness among individuals aged 11–25 in the GCC region. The campaign included culturally and religiously appropriate online mental health assessment tools, educational infographics, manuals, and videos featuring narratives from adolescents and young adults living with mental illness. Key messages such as ‘You are fine,’ ‘Be a man,’ and ‘It is just a phase’ were included in the videos to reflect community attitudes toward mental health. The video was nearly 2 min long and targeted audiences in Saudi Arabia, the UAE, Kuwait, Qatar, Oman, and Bahrain. The campaign content was shared on GHC’s official social media accounts, which have followers from the GCC and other nations. The content reached 1 million views and received almost 3,000 engagements across various social media platforms. We performed a qualitative thematic analysis of comments posted in response to the campaign on four social media platforms (TikTok, YouTube, Instagram, and X). There is diversity in the characteristics of followers based on the social media platform. For example, GHC X account followers were mainly males aged between 18 and 44, while GHC Instagram account followers were mostly females aged between 25 and 44 (17).

### Theoretical framework

Murdock’s ill-health theoretical model influenced our exploration of perceptions of mental health (18). According to Murdock, it is vital to differentiate between natural and supernatural beliefs about disease causes (18). Therefore, to better understand people’s perceptions about mental health, views on mental health could be linked to physiological or mystical causes. The importance of each definition of disease causation (natural vs. supernatural) varies across different societies, guiding our study’s design and conceptualisation.

### Data collection

**Figure 1:**
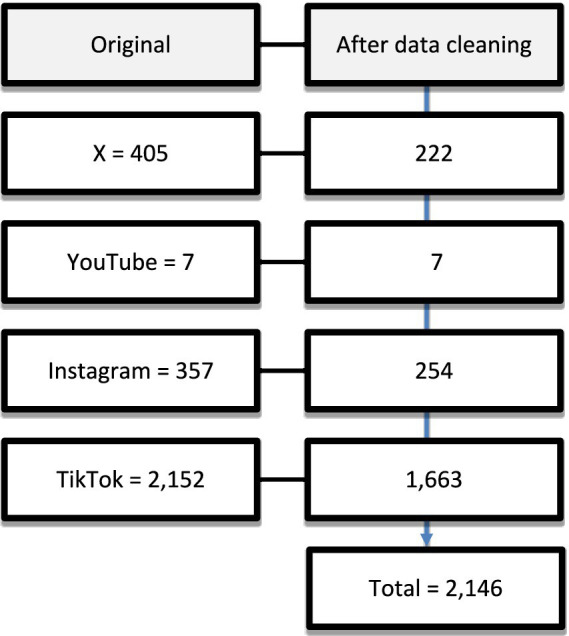
Process of cleaning datasets for qualitative analysis.

We extracted all comments on the campaign on four social media platforms (YouTube, Instagram, TikTok and X) from the 1st of September 2021 to the 10th of December 2021. We extracted all the comments posted on the campaign content from the beginning until 10 days after the last post. Posts on top social media platforms typically receive half of their total engagement (such as likes, shares, and comments) quickly, ranging from seconds to under nine days. After that half-life point, posts start to get buried in the news feed (19). Therefore, we do not expect more relevant comments to be posted ten days following the campaign. We identified 2,921 comments and extracted the data into an Excel file for data cleaning. Figure 1 details the comments included from each platform and the data cleaning process. We excluded duplicate comments, advertisements, or comments unrelated to the research aim from the analysis. After data cleaning, 2,146 comments were included in the analysis.

### Data analysis

Data was analyzed using thematic analysis (20). All comments were exported to ATLAS.ti for coding. All included comments were coded independently by SA, and a random sample of the comments from all platforms was double-coded by NA. Both coders, SA and NA, are Saudi female public health researchers experienced in qualitative research. They are trained in public health interventions and are interested in mental health research. Codes were then discussed between authors, and amendments were made in an iterative process to develop an initial coding frame. To ensure a comprehensive interpretation of the data, authors moved continuously between data familiarization, interpretations, and reporting. Once codes were refined, codes were grouped and categorized thematically to create initial themes. All quotes included in the findings were independently translated from Arabic to English by two authors (SA and NA), and then the final translation was jointly agreed upon.

### Ethical considerations

Whether data shared on social media is considered private or public remains a topic of debate (21). However, comments on a public platform could be regarded as “public data” (21). We removed identifiable information, such as usernames, from the published data to ensure ethical standards. Additionally, all quotes presented in the paper have been translated from Arabic to English, making it difficult to trace their source. We have only included comments made publicly; any data provided through direct messages or private accounts was excluded from the data.

## Results

A total of 2,146 comments were included in the analysis. Most comments were from TikTok (*n* = 1,663), followed by Instagram (*n* = 254) and X (*n* = 222). The least number of comments were from YouTube (*n* = 7). Several themes emerged from the data, including mental health is “not real,” “people have control over their mental health,” religion and mental health, the need to acknowledge mental health and strategies to promote mental health.

### Mental illness is “not real”

One of the most significant findings in the comments was how people in the GCC community define and conceptualize mental health. Denial of the existence of mental illness was evident, where mental health issues were viewed as fake illusions and negative thoughts that should be ignored.

*“The famous saying in our society is that you are fine; nothing is wrong with you.” (TikTok)*

Some comments minimized mental health issues by suggesting that negative thoughts or feeling down do not necessarily mean depression. For example, many comments mentioned that it is expected to “feel down” sometimes and that social isolation is a “personal choice” and a preference for many people.

*“Mental illness is fake illusions that only take over people who do not ignore negative thoughts. The treatment of mental illness is 90% within a person’s control. They need to fight those negative thoughts.” (TikTok)*

*“As teenagers, it’s normal that we feel depressed at this age.” (TikTok)*

Trivializing and denying the existence of mental health issues has a severe impact on people living with mental illness. The denial impacts people’s perceptions of their mental health status, which could prevent them from seeking appropriate help when needed. People tend to hide their mental health issues, fearing stigma and discrimination.

*“Hahaha, this is nonsense. Hardship is a part of life, and people must learn how to deal with it. The most important thing is positive thinking because negative thoughts will bring them down, and that’s why they feel bad. Just stay positive!” (X)*

*“You need to take people who have mental illness seriously. You need to feel for them and understand their suffering. Please do not ignore them or stigmatise them. It would be best if you stood by them… They suffer in silence.” (TikTok)*

Mental illness was believed to be a “new illness” and a “trend” that recently developed and has only become an issue in recent years. Some people think that they did not experience any of the mental health issues that young people are facing today.

*“This is for teenagers in middle and high school; they act all sad and anxious, but they are not sad; it just became a trend.” (TikTok)*

Since mental illness is openly discussed in the West, some commenters linked mental health problems to Westernization, suggesting that people with mental illness are imitating Western cultures.

*“This is a Western concept that was invented to strip away youth’s confidence and give them an excuse to misbehave.” (X)*

A prevailing belief surrounding mental health disorders is that they stem from witchcraft or the influence of the “evil eye.” Individuals with mental illnesses have often been perceived as lazy or “privileged.” Additionally, there are beliefs tied to spiritual and religious elements, such as the need for protection from oneself and from the demons of both humankind and Jinn (evil spirits). Practices such as reciting the Qur’an, maintaining regular prayers and seeking solace from Allah are frequently emphasized.

*“Some of them [mental health disorders] are not real mental illness; the reason could be black magic or the evil eye.” (TikTok)*

*“Stop being so spoiled and privileged. My problems are endless, and I am still happy and content.” (TikTok)*

*“It requires reading the Quran and maintaining prayers and the supplications that protect you from yourself and the devils among humans and Jinn. It requires the night prayer and confiding in Allah Almighty. It requires optimism.” (TikTok)*

### People have control over their mental health

There is a common belief that individuals should be capable of addressing their mental health challenges and overcoming them independently. A commonly quoted comment was, “You are in control of your mental state.”

*“I see it this way, from its name, mental problems [personal issues in literal translation from Arabic]. It means it is personal, and the person should be able to solve it.” (TikTok)*

Mental illness is believed to be caused by people’s inability to cope with life’s challenges. Facing challenges is a normal part of living, and people should be able to cope with life issues such as financial difficulties, unemployment, and difficulties with their studies. Learning to deal with life with a “positive attitude” was perceived to be the solution to dealing with mental health problems.

*“Life stresses have always existed and will always exist; that’s the nature of life. The important thing is that you keep a positive attitude and avoid negative thoughts. Negative thoughts are the cause of mental health issues. Just be positive!” (X)*

Mental illness was often viewed as a sign of weakness, particularly in terms of masculinity. Commenters usually associate mental health issues with a lack of manliness. According to some comments, individuals who are considered “real men” do not have mental illness, as it is seen as a sign of weakness and a lack of control over one’s emotions.

*“When a boy goes to his father and says I have an illness and I need help, he would say be a man! There’s nothing wrong with you!” (TikTok)*

The use of physical punishments with children, specifically male children, was viewed positively as a way to raise strong men capable of controlling their emotional and mental well-being.

*“Our parents used to break the wooden stick on our backs [hit us], and we grew up fine, not knowing depression or mental illness. This is the problem when you grow up privileged and weak!” (TikTok)*

### Religion and mental health

Numerous comments discussed the relationship between mental health and religion. A common perception is that the cause of mental illness is a lack of relationship with God and spiritual failings. Religion and spirituality were commonly believed to be the best ways to prevent and treat mental illness. People must focus on their relationship with God and improve their commitment to religious practices, such as praying and reading the Quran daily to promote mental health.

*“I swear to God, if the person prays and reads the Quran and loves God, God will not make them depressed, regardless of their situation.” (TikTok)*

*“Your relationship with God will determine your mental health status. When you are not religious, your mental health will deteriorate, and you will feel lost and living your life without purpose.” (TikTok)*

Many people associate mental illness with moral and spiritual shortcomings, which can make those with mental health issues afraid of being perceived as irreligious. It was observed that people living with mental health issues were trying to defend their religiosity in the comments. People with mental health disorders tried to explain that anyone can experience mental illness regardless of their religious beliefs or practices.

*“I am so exhausted from feeling depressed, I am struggling. Although I pray and read the Quran.” (TikTok)*

Some people believe that those with mental illness deserve their suffering, as they associate it with turning away from God; some people mention that people with mental illness *“deserve their suffering”* as *“whoever turns away from God will live a miserable, sad life*.”

### The need to acknowledge mental health

Many commenters mentioned that for people to be able to seek help for a mental illness, society needs to acknowledge its existence as an actual medical condition. Comments explained that it would be easy for people to respond and seek medical care for a physical illness but not a mental illness.

*“Mental health is just as important as physical health. When you have a headache, acknowledging it and taking medication for it is easy. However, when it comes to mental illness, so many people are lost and suffering in silence.” (X)*

*“We need to take care of our mental health just as we take care of our physical health.” (Instagram)*

Society’s denial of mental health issues was believed to contribute to the increase in mental health disorders and exacerbate mental illness. People living with mental health problems discussed the difficulty of explaining what they feel or experience. The difficulty is mainly due to a lack of community understanding and sympathy for people with mental health problems. This lack of compassion and understanding extends beyond society and is often experienced by their family and close friends.

*“The issue is that as much as you try to explain and communicate what you are experiencing, no one will understand you, no one will sympathise.” (TikTok)*

*“Everyone in the comments is suffering from depression, and I do not blame you since you live in an Arab society.” (TikTok)*

### Strategies to promote mental health

Several strategies and solutions were proposed to acknowledge and promote mental health in the GCC. This includes efforts to raise mental health awareness in the GCC community, which were praised in some comments. To promote mental health, many comments highlighted the need to have an open dialogue about mental health in the community.

*“Please continue to produce content that spreads awareness about the reality of mental illness and how people can prevent it.” (Instagram)*

*“I wish there’s more awareness of the importance of mental health promotion in our societies.” (TikTok)*

*“We need more of this type of awareness. Finally, someone is talking about mental health.” (TikTok)*

Some commenters criticized the images and videos used in mental health campaigns, where mental illness was often depicted in dark colors and depressing content. Therefore, some commenters suggested that the colors and depiction of mental health must portray positivity and optimism.

*“Please avoid dark colours in the design of awareness content relating to mental health. The content should be presented in light colours to promote positivity.” (X)*

The comments stressed the crucial role of creating content that reflects real-life experiences and situations to gain influence and be effective. Content that mirrors reality can resonate more deeply with audiences, potentially leading to more significant impact and success.

*“I suffer from mental illness. The video resonated with me; I feel it speaks to me.” (TikTok)*

An important issue highlighted by some commenters was the lack of strategies that promote mental health that goes beyond awareness. Appropriate interventions to promote mental well-being were viewed as necessary yet lacking. Removing barriers to seeking treatment and reducing the stigma attached to mental illness is a crucial step toward mental health promotion.

*“We are thrilled that mental health is starting to be acknowledged and is focused on in the GCC. I hope we start to use our resources to develop programs that raise awareness and encourage people to seek treatment for the long term.” (Instagram)*

Concerns were expressed when using online tools and surveys to assess mental health, as some people may rely on them instead of seeking help from a qualified professional.

*“These tools do not diagnose depression. A psychiatrist diagnoses mental illnesses, not a channel or a quiz.” (TikTok)*

The need to start mental health awareness in schools was expressed in the comments. Improving mental health awareness in schools was believed to lead to overall improvements in the mental health of younger generations and prevent avoidable mental health disorders.

*“It is essential to develop programs to reduce and prevent mental health disorders before they get worse for the young generation. We need to work on promoting the mental health of students in schools, especially since they represent a large segment of our society.” (X)*

## Discussion

This study explored people’s perceptions and views of a mental health social media campaign targeting adolescents in the GCC. Our findings highlight significant misconceptions and stigmas surrounding mental health in the GCC community. Mental illness is often believed to be caused by witchcraft or physical illness and is associated with poor religious beliefs. Dealing with mental health issues is perceived as a weakness and lack of willpower. Coping with mental illness is believed to be within the person’s control, and having a positive attitude is seen as the solution. The lack of strategies to promote mental health and the stigma attached to mental illness can hinder seeking appropriate medical care.

Comments revealed significant stigma surrounding mental illness. The negative impact of mental health stigma has been well documented and described by those with mental illness as ‘worse than the illness itself’ (22). Negative attitudes and stigma toward mental illness result from a complex set of factors (11). Stigma toward mental illness can be influenced by poor health literacy, lack of mental healthcare services, cultural perspectives, and religious beliefs. Research revealed that Muslims have lower mental health literacy compared to non-Muslims (23). However, recent studies show significant improvements in mental health literacy and considerable reductions in mental health stigma in certain countries and for specific mental health conditions (24, 25). These reductions have been reportedly linked to improvements in education, healthcare infrastructure, accessibility, and high levels of exposure to mental health campaigns in recent years (26–28).

Our findings revealed that a prevalent belief was that supernatural forces, such as witchcraft or the evil eye, could cause mental illness. This belief aligns with existing literature on the cultural context of mental health in the GCC, where mental illness is often attributed to external spiritual influences, such as Jinn (evil spirits) or black magic (26, 29). This belief reflects a significant cultural context that shapes public perceptions of mental health within the GCC. Such perceptions further complicate the recognition of mental health disorders as legitimate medical conditions and contribute to stigma (26, 29). These findings underscore the importance of addressing these cultural beliefs when developing mental health interventions, as they can influence both the willingness to seek professional help and the way mental health issues are perceived by society (4).

Religious coping emerged as a prominent theme in the comments, with many participants attributing their mental health resilience to religious practices. It is also well-documented that many conservative communities believe that mental illness is a punishment from God for moral shortcomings (6, 29, 30). This poses a significant public health challenge as it can hinder the recognition of medical or psychiatric issues by patients and their family members (26, 29, 31). The association of mental illness with evil spirits and moral failings often lead to individuals with mental illness being ostracized from society and subjected to harmful traditional practices believed to treat mental illness (26, 32).

Religious beliefs play a significant role in shaping people’s attitudes toward mental illness (7). Consistent with our findings, many Muslims often prefer seeking religious guidance over professional help (4, 23, 33). A study comparing the attitudes toward mental health between young British Muslims and non-Muslims found that over one-third of Muslims in their sample would consider stopping their psychotropic medication if advised by a non-medically trained religious leader (23). This emphasizes the critical need to consider spiritual and cultural sensitivity when developing interventions targeting mental health within Muslim communities.

### Implications for research, policy and practice

Despite the Arab countries representing 5.54% of the global population, the mental health research output accounts for 1.0% of the worldwide peer-reviewed publications (34). The lack of mental health research can hinder the development of effective treatments and interventions for mental health disorders. Therefore, there is an urgent need for increased research efforts to address the burden of mental illness in the Arab region and facilitate the improvement of the overall well-being and quality of life of individuals affected by mental disorders.

Some commenters highlighted a lack of strategies that go beyond raising awareness. While awareness is essential, interventions that promote mental well-being, focusing on stigma reduction and acceptance of mental health disorders, are necessary. This can be a crucial step toward improving mental health promotion. Ensuring people can access appropriate interventions to help them lead healthy lives and maintain their mental well-being is essential. This will require a concerted effort from individuals, healthcare providers, policymakers, and religious leaders to develop and implement effective strategies to address this issue.

Addressing mental health stigma requires a comprehensive approach that considers cultural, religious, and societal factors and promotes health literacy, education, and support (35). Anti-stigma interventions are vital for promoting positive attitudes, improving mental health literacy, and potentially reducing public and self-stigma associated with mental illness (36). Integrating mental health education in schools has been proven effective in reducing mental health stigma and improving help-seeking behaviors (36, 37). Collaboration between mental health professionals and religious leaders can facilitate addressing misconceptions and encourage support and treatment for individuals with mental illness (38, 39). Integrating mental health services within primary care settings has the potential to improve accessibility to mental healthcare and reduce the stigma associated with seeking mental health support (40, 41).

The connection between mental illness and supernatural causes remains a central theme in the cultural context of the region (4). This suggests that while mental illness may not always be directly linked to Jinn (evil spirits) in individual cases, such beliefs still hold sway in shaping attitudes toward mental health, underscoring the need for culturally tailored mental health education and intervention strategies.

### Strengths and limitations

This study presents unique findings based on the methodology used. To our knowledge, this is the first study that examines the public’s reactions to mental health awareness content in a neutral environment in the GCC. By analyzing comments posted online, we aimed to minimize the impact of social desirability bias, as these comments reflect the honest opinions of the commenters. We have included all the comments posted in response to the campaign on four different social media platforms, which means we have captured the thoughts of people from various countries in the GCC and beyond.

Due to the nature of social media platforms, we do not have demographic data about the commenters, such as age, gender, and geographical location. Social media’s anonymity increases the chance of encountering negative or unfavorable comments. This, in turn, may lead to an overrepresentation of negative viewpoints that may not accurately reflect the beliefs of the GCC community (17). Social media comments are often short and may require more in-depth analysis than other narratives to understand their meaning fully. Due to the need for more contextual information, exploring the underlying message of comments can be challenging.

Because the data is in written form, misinterpretation is possible. To address this challenge, the researchers held continuous meetings and discussions to clarify any uncertainties in the data. They have also included as many quotes as possible from the data to improve trustworthiness, maximize validity, and enhance the reader’s interpretation.

A noted limitation of this study is the need for more triangulation. Future research should consider incorporating qualitative interviews or focus groups to gain deeper insights into the perceptions of mental health beyond online comments, particularly given the complex interplay of cultural and religious factors in the GCC.

## Conclusion

Our research highlights the lack of awareness, stigma, and insufficient resources and support for individuals dealing with mental health issues. This sheds light on the importance of addressing the barriers to mental healthcare and increasing access to resources and support for those who need it. Interventions that raise awareness and focus on reducing stigma and promoting acceptance of mental health disorders are crucial. This requires a joint effort from individuals, healthcare providers, policymakers, and religious leaders to develop and implement effective strategies.

## Author’s note

We confirm that we have thoroughly read and understood the terms of use for TikTok, YouTube, Instagram, and X regarding social media research. We ensured that our study adhered to all relevant policies and standards.

## Data Availability

The original contributions presented in the study are included in the article/supplementary material, further inquiries can be directed to the corresponding author.
