# Corrigendum: Public perceptions of digital mental health awareness campaign in the Arab Gulf states: a qualitative thematic analysis

**DOI:** 10.3389/fpubh.2025.1597875

**Published:** 2025-05-14

**Authors:** Noura Alomair, Ghadah Alkhaldi, Norah M. Alsadhan, Rawan Alkasabi, Samah Alageel

**Affiliations:** ^1^Department of Community Health Sciences, College of Applied Medical Sciences, King Saud University, Riyadh, Saudi Arabia; ^2^Insurance Operations Policies Department, Insurance Authority, Riyadh, Saudi Arabia

**Keywords:** mental health, digital health, mental illness, culture, religion, stigma

In the published article, the reference for (19) was incorrectly written as “GRAFFIUS SM. Half-life for posts on different social media platforms”. It should be:

19. Graffius SM. Lifespan (half-life) of social media posts: update for 2024. (2024). doi: 10.13140/RG.2.2.21043.60965

The published article erroneously paraphrased the lifespan of social media posts.

A correction has been made to **Methods**, *Data collection*. This sentence previously stated:

“The lifespan of social media posts on most platforms is, at most, 9 days.”

The corrected sentence appears below:

“Posts on top social media platforms typically receive half of their total engagement (such as likes, shares, and comments) quickly, ranging from seconds to under nine days. After that half-life point, posts start to get buried in the news feed.”

The authors apologize for this error and state that this does not change the scientific conclusions of the article in any way. The original article has been updated.

